# A Cough Deteriorating Gross Hematuria: A Clinical Sign of a Forthcoming Life-Threatening Rupture of an Intraparenchymal Aneurysm of Renal Artery (Wunderlich's Syndrome)

**DOI:** 10.1155/2013/452317

**Published:** 2013-06-20

**Authors:** Ioannis Anastasiou, Ioannis Katafigiotis, Christos Pournaras, Evangelos Fragkiadis, Ioannis Leotsakos, Dionysios Mitropoulos, Constantinos A. Constantinides

**Affiliations:** Department of Urology, Laiko Hospital, University of Athens, Greece

## Abstract

Macroscopic hematuria regards the 4% to 20% of all urological visits. Renal artery aneurysms (RAAs) are detected in approximately 0.01%–1% of the general population, while intraparenchymal renal artery aneurysms (IPRAAs) are even more rarely detected in less than 10% of patients with RAAs. We present a case of a 58-year-old woman that came into the emergency room (ER) complaining of a gross hematuria during the last four days. Although in the ER room the first urine sample was clear after a cough episode, a severe gross hematuria began which led to a hemodynamically unstable patient. Finally, a radical nephrectomy was performed, and an IPRAA was the final diagnosis. A cough deteriorating hematuria could be attributed to a ruptured intraparenchymal renal artery aneurysm, which even though constitutes a rare entity, it is a life-threatening medical emergency.

## 1. Introduction 

Macroscopic hematuria is a medical condition that the urologist frequently must face in the emergency room (ER) and constitutes the 4% to 20% of all urological visits [1]. Even the most serious cases of gross hematuria that require hospital admission of the patient usually are not life-threatening and in the worst-case scenario can be managed with blood transfusion and perhaps a transurethral operation later in time if the cause is located in the prostate gland or the bladder. Wunderlich's syndrome is a spontaneous nontraumatic bleeding confined to the subcapsular and/or perinephric spaces in patients with no known underlying cause and constitutes an emergency medical condition [2, 3]. Renal artery aneurysms (RAAs) are detected in approximately 0.01%–1% of the general population, while intraparenchymal renal artery aneurysms (IPRAAs) are even more rarely detected in less than 10% of patients with RAAs [4]. We present a case of a 58-year-old woman that presented in the emergency room of the urology clinic complaining of intermittent gross hematuria but finally became hemodynamically unstable due to rupture of an intraparenchymal aneurysm of the renal artery, and a radical nephrectomy in an emergency basis was performed and saved her life. 

## 2. Case Presentation 

**Figure 1:**
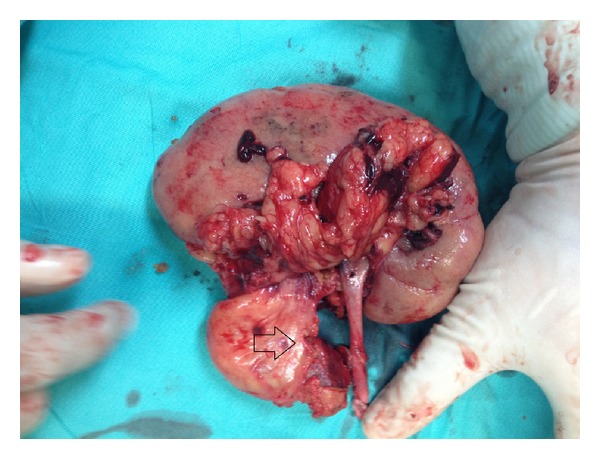
An intraparenchymal renal artery aneurysm.

**Figure 2:**
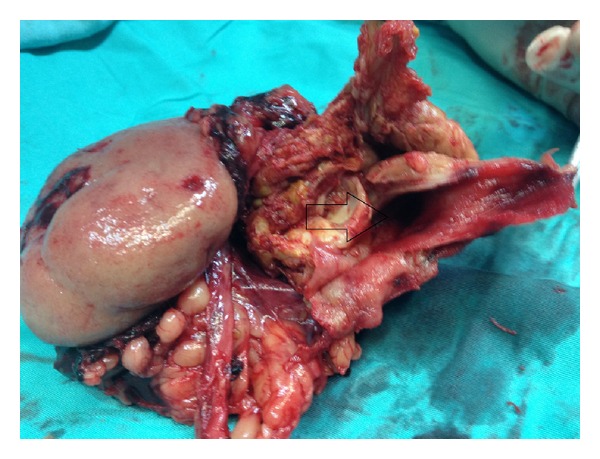
An intraparenchymal renal artery aneurysm.

**Figure 3:**
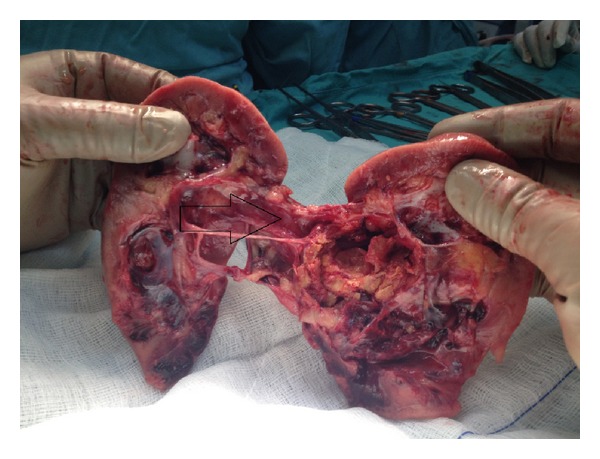
An intraparenchymal renal artery aneurysm.

A 58-year-old woman presented in the ER of the urology clinic complaining of intermittent gross hematuria during the last four days and a mild flank pain during the last hour. The patient also mentioned that the hematuria was deteriorating only during defecating. Arterial hypertension was her only concomitant medical condition. The first sample of urine that we obtained was macroscopically normal with positive dipstick for blood in the urine. Her blood pressure in the ER room was 130/80 mmHg and the heart rate 75/min. The first blood examination showed a 38% hematocrit with 12 hemoglobin and normal urea and creatinine. The ultrasound of the kidneys and the bladder showed a solid formation in the upper pole of the right kidney with peripheral blood flow, while in the bladder, solid mobile formations were present as in blood clots. The second sample of urine that we obtained had macroscopically intense hematuria, and we inserted a three-way catheter performing at first manual washouts with normal saline of the bladder clearing the blood clots and adjusting an irrigating normal saline finally. While waiting for the computed tomography to be performed, the patient had clear urine without blood, and we stopped the irrigation fluid in order to examine if the urine remained cleared without the washouts. The urine remained clear, but after a cough episode, instantly the urine became intense bloody and the patient became hemodynamically unstable, while the blood pressure reduced steadily from 130/80 to 60/50 mmHg, the heart rate increased from 75/min to 140/min, and the pulse was detectable only in a central artery. The hematocrit reduced also steadily from 38% to 33% and till 18%. The patient was driven urgently in the operation room, and since a definite diagnosis was not made, both a surgeon urologist and a vascular surgeon participated in the operation. An exploratory laparotomy was performed with a midline incision in the search of a possible aortic aneurysm. Finally, a large ruptured intraparechymal aneurysm of the right renal artery was detected and a radical nephrectomy was performed (Figures 1, 2, and 3). The first two postoperative days the patient was admitted to the intensive unit of our hospital and then to the urology clinic from which she was discharged 8 days later.

## 3. Discussion 

Gross hematuria can occur in up to 2.5% of the population [5]. The most common reasons of gross hematuria are urolithiasis, infectious diseases, and carcinoma of the urinary tract [6, 7]. The majority of the patients with gross hematuria is managed conservatively in an outpatient basis, and strict instructions are given to the patient both for the management and the followup of the hematuria given the fact that macroscopic hematuria has a high diagnostic yield for urological malignancy [6–9]. The most important factor is the macroscopic hematuria to resolve with adequate water consumption and the blood examinations to show a stable hematocrit which is combined with a stable hemodynamically condition, and if there is no possibility of a urinary retention from a blood clot, then usually the patient is discharged with certain instructions and examinations that should be performed in order to rule out the possibility of cancer. 

In the emergency room, a complete set of examinations including urine test, blood count, urea, creatinine, and electrolytes levels count and also a basic radiologic imaging such as an ultrasound and an X-ray of the kidneys, ureters, and bladder are usually performed in order to examine the possible cause of the hematuria and of course its severity [9]. Patients with a stable hemodynamic condition without a cardiovascular compromise, with no evidence of acute renal failure, sepsis, and clot retention, without inability to take adequate oral fluids, serious comorbidity or social circumstances requiring admission, outpatient management can be considered [9]. In this base and considering the fact that usually gross hematuria is due to easily managed medical conditions combining with the fact that a rupture of an aneurysm is uncommon [10], the patient is usually discharged constituting an intermittent hematuria caused by an IPRAA, an easily missed diagnosis that could seriously compromise the life of the patient. Wunderlich's syndrome is a dreaded life threatening complication of a ruptured IPRAA that can be fatal if not treated promptly and aggressively [11, 12]. RAAs are rare and constitute localized dilations of the renal artery and/or branches [4, 13]. RAAs can be divided into 4 basic categories: the saccular, fusiform, dissecting, and the arteriovenous/microaneurysms (intrarenal) [10, 13, 14]. RAAs constitute the 22% of visceral aneurysms, and the most common RAAs are the saccular representing 70% to 75% of all RAAs, while the IPRAAs are a rare entity representing the 10% of all RAAs [10, 13, 14]. The mean age at diagnosis is 60 years, and RAAs are usually located in the right kidney as in our case [13]. Predisposing factors for RAAs are congenital malformations of the kidney (congenital type of RAAs), untreated hypertension, atherosclerosis, pregnancy, trauma, recent surgical manipulation, malignancy, renal angiomyolipoma, radiation, and/or cyclophosphamide use (acquired type of RAAs) [10, 13, 14]. In our case, the female patient had only hypertension from her medical history but not untreated, and no trauma or recent surgical manipulation was reported, making the diagnosis of an RAA even more difficult. RAAs are frequently asymptomatic, and main complications include rupture, thrombosis, distal emboli, renovascular hypertension, intrarenal aneurysm, and arteriovenous fistulas [13, 14]. 

Rupture is the most feared complication and the risk depends on the age, the gender, the histology, and the size of the aneurysm [13, 14]. Rupture of an RAA is a rare event and severe gross hematuria due to the fact that rupture of an IPRAA into the renal pelvis is even more rare make the diagnosis in the ER difficult at least for the inexperienced resident in urology [10, 13]. Symptoms like flank pain and hematuria ranging from microscopic to severe gross hematuria and subsequently to shock and hemodynamic instability are general symptoms that occur in a rupture of an RAA and usually are not very helpful in the definite diagnosis of an RAA in the ER room. An important symptom that could help to the diagnosis of a possible forthcoming IPRAA rupture is the deterioration of the gross hematuria with the increase of the intra-abdominal pressure as during defecating or coughing as in our case. Endovascular surgery and especially transcatheter arterial (TAE) embolization are considered to be the treatment of choice for the management of RAAs [4, 13]. TAE in many cases is the treatment of choice even in the emergency situation of an RAA rupture [13]. Of course in our case, the rupture caused a life threatening hemorrhage and shock which combined with the fact that we did not have a definite diagnosis open exploratory laparotomy, and subsequently a radical nephrectomy was the only choice. 

## References

[1] Mariani AJ, Mariani MC, Macchioni C, Stams UK, Hariharan A, Moriera A (1989). The significance of adult hematuria: 1,000 hematuria evaluations including a risk-benefit and cost-effectiveness analysis. *The Journal of Urology*.

[2] Vaddi SP, Reddy VP, Devraj R (2011). Wunderlich’s syndrome in a tuberous sclerosis patient. *Indian Journal of Surgery*.

[3] Medda M, Picozzi SC, Bozzini G, Carmignani L (2009). Wunderlich's syndrome and hemorrhagic shock. *Journal of Emergencies, Trauma, and Shock*.

[4] Porcaro AB, Migliorini F, Pianon R (2004). Intraparenchymal renal artery aneurysms. Case report with review and update of the literature. *International urology and nephrology*.

[5] Ritchie CD, Bevan EA, Collier St. J (1986). Importance of occult haematuria found at screening. *British Medical Journal*.

[6] Antoniewicz AA, Zapała Ł, Poletajew S, Borówka A (2012). Macroscopic hematuria—a leading urological problem in patients on anticoagulant therapy: is the common diagnostic standard still advisable?. *ISRN Urology*.

[7] Wallace DMA, Bryan RT, Dunn JA, Begum G, Bathers S (2002). Delay and survival in bladder cancer. *BJU International*.

[8] Thiruchelvam N, Mostafid H (2005). Do patients with frank haematuria referred under the two-week rule have a higher incidence of bladder cancer?. *Annals of the Royal College of Surgeons of England*.

[9] Hicks D, Li C-Y (2007). Management of macroscopic haematuria in the emergency department. *Emergency Medicine Journal*.

[10] De Wilde V, Devue K, Vandenbroucke F, Breucq C, De Maeseneer M, De Mey J (2007). Rupture of renal artery aneurysm into the renal pelvis, clinically mimicking renal colic: diagnosis with multidetector CT. *The British Journal of Radiology*.

[11] Fraser GE, Poncia H (2009). Spontaneous renal artery aneurysm rupture: an unusual cause of abdominal pain and syncope. *Emergency Medicine Journal*.

[12] Eskandari MK, Resnick SA (2005). Aneurysms of the renal artery. *Seminars in Vascular Surgery*.

[13] Shaun EL, Wason MD (2010). Spontaneous rupture of a renal artery aneurysm presenting as gross hematuria. *Reviews in Urology*.

[14] Özkan G, Ulusoy S, Dinç H, Kaynar K, Sönmez B, Akagündüz K (2011). Bilateral asymptomatic giant renal artery aneurysm. *Hippokratia*.

